# A Novel Aggregation-Induced Emission Fluorescent Probe for Detection of β-Amyloid Based on Pyridinyltriphenylamine and Quinoline–Malononitrile

**DOI:** 10.3390/bios13060610

**Published:** 2023-06-02

**Authors:** Yan Fang, Qi Wang, Chenlong Xiang, Guijin Liu, Junjian Li

**Affiliations:** Key Laboratory of Tropical Biological Resources of Ministry of Education, School of Pharmaceutical Sciences, Hainan University, Haikou 570228, China; 20086000210007@hainanu.edu.cn (Y.F.); 21220860000025@hainanu.edu.cn (Q.W.); 20192114310019@hainanu.edu.cn (C.X.)

**Keywords:** aggregation-induced emission fluorescent probe, β-amyloid, viscosity, Alzheimer’s disease

## Abstract

β-amyloid is an important pathological feature of Alzheimer’s disease. Its abnormal production and aggregation in the patient’s brain is an important basis for the early diagnosis and confirmation of Alzheimer’s disease. In this study, a novel aggregation-induced emission fluorescent probe, PTPA-QM, was designed and synthesized based on pyridinyltriphenylamine and quinoline–malononitrile. These molecules exhibit a donor–donor–π–acceptor structure with a distorted intramolecular charge transfer feature. PTPA-QM displayed the advantages of good selectivity toward viscosity. The fluorescence intensity of PTPA-QM in 99% glycerol solution was 22-fold higher than that in pure DMSO. PTPA-QM has been confirmed to have excellent membrane permeability and low toxicity. More importantly, PTPA-QM exhibits a high affinity towards β-amyloid in brain sections of 5XFAD mice and classical inflammatory cognitive impairment mice. In conclusion, our work provides a promising tool for the detection of β-amyloid.

## 1. Introduction

With the further improvement of the economic standard of living and the intensification of an aging society, Alzheimer’s disease (AD), Parkinson’s disease (PD), and other neurodegenerative chronic diseases have become a major problem that threatens the health of the general public [1,2,3,4,5]. AD is a common clinical degenerative disorder of the central nervous system, which can lead to memory impairment, aphasia, visuospatial impairment, and executive dysfunction [6]. Currently, the common clinical diagnostic tools for AD rely on clinical symptoms combined with imaging, including computed tomography, magnetic resonance imaging, and positron emission tomography [7,8]. These methods are cumbersome, of low specificity, have a short half-life, are expensive, and have unavoidable limitations due to radiation risks to humans [9]. Therefore, there is an urgent need to develop a highly sensitive, non-invasive, and safe diagnostic imaging technique. 

The currently recognized pathogenesis of AD consists of five main aspects: amyloid peptides (Aβ) deposition and toxic effects, Tau protein hyperphosphorylation, cholinergic neuronal deficits, excitatory amino acid toxicity, and neuroinflammation [10,11,12]. In addition, neuroinflammation can induce neuronal tangles and Aβ deposition, which in turn mediates neurotoxicity and neuronal apoptosis, resulting in patients exhibiting varying degrees of cognitive dysfunction [13,14]. Therefore, Aβ, as a key pathological target, is important for the detection and therapeutic research of neurodegenerative diseases [15,16,17,18]. Aβ is a 4 kD small peptide fragment obtained by the sequential hydrolysis of β-amyloid precursor protein using protein hydrolases β and γ. It mainly includes Aβ1-40 and Aβ1-42, where Aβ1-42 is the initiator and main structural substance of senile plaque and can have toxic effects on neuronal cells [19,20]. The fluorescence imaging technique has the advantages of safety and simplicity, low cost, good real-time performance, and high sensitivity [21]. Therefore, it is a safe and non-destructive imaging technique. It is well suited for Aβ detection applications. Currently, Congo red (CR) derivatives and thioflavin T (ThT) derivatives are commonly used fluorescent probes for the detection of amyloid plaques [22,23,24]. It can specifically identify Aβ fibers. However, there are also non-negligible drawbacks. For example, the Stokes shift is small, and the specificity and sensitivity are poor [25]. When binding to Aβ fibers, it tends to accumulate π-π and leads to aggregated fluorescence burst (ACQ), which significantly reduces the detection efficiency. It even causes signal loss or “false positive” results. Therefore, the research and development of novel small molecule fluorescent probes for Aβ plaques is particularly urgent. 

**Figure 1:**
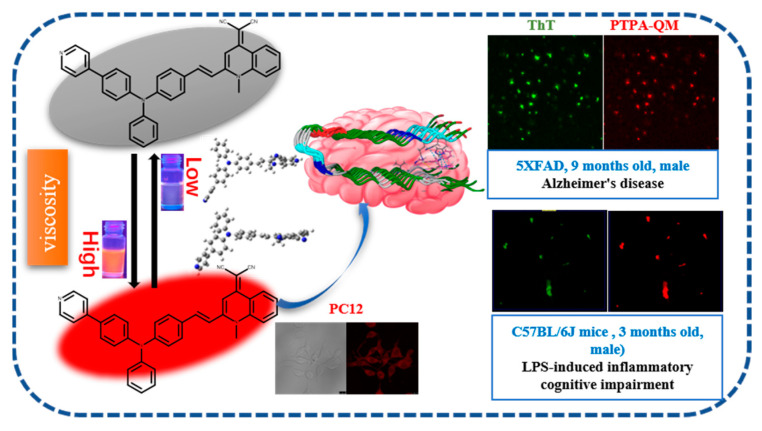
Schematic diagram of PTPA-QM monitoring of viscosity fluctuations and Aβ images in different mouse models.

Compared to conventional organic dyes, aggregation-induced emission (AIE) fluorescent probes have large Stokes shifts, strong fluorescence, and good resistance to bleaching [26,27,28,29,30]. The chromophore quinoline–malononitrile (QM) has recently emerged as a novel AIE building block with some remarkable features, such as red to near-infrared (NIR) emission, high brightness, remarkable photostability, and good biocompatibility [31,32,33]. Thus, the long-wavelength fluorescence emission of QM has made it a useful chemical tool for imaging disease-related biomarkers in cells and in vivo [34,35,36]. Herein, we set out to AIE the fluorescent probes PTPA-QM (Figure 1) employing the QM and triphenylamine aldehyde derivative as the AIE building block. The constructed probe, PTPA-QM, has good optical activity. In low-viscosity solutions, it has a very weak fluorescence due to the intramolecular distorted internal charge transfer (TICT) effect between the α,β-unsaturated bonds. With the increase in solution viscosity, the emission of red wavelengths is greatly enhanced. In addition, owing to the low cytotoxicity and excellent photostability, PTPA-QM can be used for fluorescence imaging with PC12 cells. To our delight, PTPA-QM can image Aβ in the brain of 5XFAD mice and classical inflammatory cognitive impairment mice. Our AIE probe holds great promise for exploring the early diagnosis of diseases caused by Aβ.

## 2. Experimental Section

### 2.1. Materials and Methods

All the reagents and solvents used were analytical grade and obtained commercially without further purification. Ultrapure water was used throughout the experiment. Various biologically relevant species (Ca^2+^, Fe^3+^, K^+^, Na^+^, F^−^, Cl^−^, Br^−^, I^−^, SO_3_^2−^, CO_3_^2−^, HSO_3_^−^, H_2_O_2_, GSH, TrP, Phe, Met, Thr, Glu, Lys) were purchased from Sigma-Aldrich. ^1^H NMR and ^13^C NMR spectra were carried out on 400 MHz and 125 MHz, and TMS was used as an internal standard. A Shimadzu UV-2550 spectrometer was performed to obtain UV-vis spectra. Fluorescent spectra were achieved using a Hitachi F-4700 fluorescence spectrophotometer. The probe PTPA-QM solid was prepared with dimethyl sulfoxide (DMSO) solution to form a 1 × 10^−3^ mol/L probe master mix. The master mix was placed in a refrigerator and set aside. The above PTPA-QM solution was diluted to the desired solution concentration. A complete cytotoxicity assay using PC12 cells and labeling using brain sections are provided in the Supporting Information.

### 2.2. Synthesis of Compound PTPA-QM

**Scheme 1:**
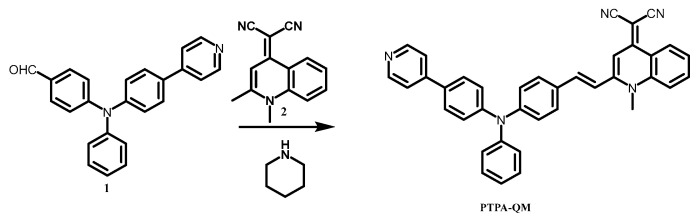
The synthesis routine of PTPA-QM.

The whole synthesis routine is provided in Scheme 1. Compound **1** (351 mg, 1 mmol) was dissolved with Compound **2** (221 mg, 1 mmol) in anhydrous acetonitrile, and 0.5 mL of piperidine was added and refluxed for 12 h. At the end of the reaction, a clear precipitate could be observed, filtered, and washed with a small amount of acetonitrile. The compound PTPA-QM was recrystallized to obtain a brown–red solid as the desired product (0.2560 g, yield: 46%).

^1^H NMR (400 MHz, DMSO) δ 8.91 (d, *J* = 8.3 Hz, 1H), 8.60 (d, *J* = 5.9 Hz, 2H), 8.04 (s, 1H), 7.93 (s, 1H), 7.79 (d, *J* = 8.6 Hz, 2H), 7.75 (d, *J* = 8.6 Hz, 2H), 7.68 (d, *J* = 5.9 Hz, 2H), 7.62 (s, 1H), 7.46 (s, 1H), 7.41 (d, *J* = 8.3 Hz, 2H), 7.39 (s, 1H), 7.19 (s, 1H), 7.15 (dd, *J* = 8.2, 3.0 Hz, 4H), 7.07 (d, *J* = 8.5 Hz, 2H), 7.03 (s, 1H), 4.00 (s, 3H). ^13^C NMR (101 MHz, CDCl_3_) δ 153.33, 150.48, 149.36, 149.07, 148.11, 147.58, 146.65, 139.63, 139.47, 133.36, 132.79, 129.94, 129.17, 128.96, 128.14, 126.60, 126.04, 124.93, 124.85, 124.71, 123.05, 121.38, 121.20, 120.51, 119.41, 117.99, 116.59, 107.51, 50.91, 37.17. HRMS (ESI-TOF) Calcd for C_38_H_27_N_5_ [M+H]^+^: 554.2320.

## 3. Results and Discussion

### 3.1. Design and Synthesis of Probe PTPA-QM

The fluorescent probe PTPA-QM was successfully prepared according to the synthetic path shown in Scheme 1. The structure was confirmed by ^1^H NMR, ^13^C NMR, and HRMS. The Knoevenagel condensation reaction was used to form a fluorescent probe with a donor–donor–π–acceptor (D1-D2-π-A) from a triphenylamine aldehyde derivative and a quinoline–malononitrile derivative with a good power supply and large conjugation system. Triphenylamine derivatives were used as the electron-donating group and quinoline as the electron-absorbing group, which were linked by ethylene bonds to construct fluorescent probes.

### 3.2. Photophysical Properties of PTPA-QM

**Figure 2:**
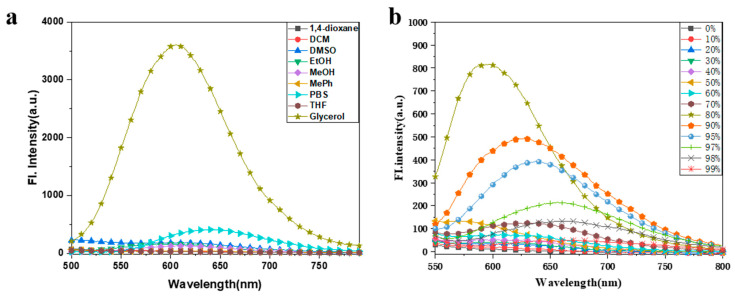
Fluorescence emission spectra of PTPA-QM (**a**) in different solvents and (**b**) in PBS-DMSO mixture with different DMSO volume fractions (10 μM).

In order to investigate whether PTPA-QM has a solvent effect, the fluorescence spectra of PTPA-QM in different solvents were investigated. As shown in Figure 2a, in a low-viscosity solution PBS, PTPA-QM has a weak fluorescence emission peak at 645 nm. In the glycerol system with high-viscosity PTPA-QM, the red fluorescence was significantly enhanced at 605 nm. The maximum absorption peaks were located at 448 nm. The fluorescence intensity increased about eight-fold. PTPA-QM in other organic solvents with different polarities was negligible compared to the strong fluorescence in glycerol. The quantum yield of the probe PTPA-QM was 0.09% in THF and 3.93% in glycerol. Therefore, PTPA-QM could serve as an effective probe to detect changes in the viscosity.

Subsequently, the fluorescence spectra of the fluorescent probe PTPA-QM were examined in different volume ratios of PBS mixed with DMSO. As can be observed from Figure 2b, a weak emission peak of PTPA-QM at 645 nm was observed when the volume fraction of water was less than 60%. When the volume fraction of PBS (fw) is greater than 60%, the fluorescence intensity shows a significant enhancement. While the fluorescence intensity reaches a maximum in PBS / DMSO (4:1 = *v*/*v*), the emission wavelength shows a certain degree of blue shift, indicating the gradual formation of nanoaggregates. However, the fluorescence intensity increased about 12-fold. It is demonstrated that the fluorescent probe PTPA-QM is a molecule with typical AIE properties, and that the intramolecular rotation of compound PTPA-QM is restricted in the aggregated state, thus causing intense fluorescence emission. In addition, this is consistent with the color change visible to the naked eye under UV light, which fluoresces faintly at first and eventually produces a red fluorescence.

**Figure 3:**
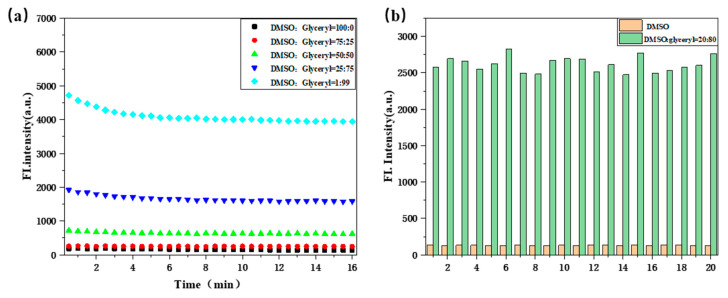
(**a**) Time-dependent fluorescence intensity changes in PTPA-QM (10 μM) in the DMSO:glycerol = 100:0, DMSO:glycerol = 75:25, DMSO:glycerol = 50:50, DMSO:glycerol = 25:75, and DMSO:glycerol = 1:99, *v*/*v*. (**b**) The change in fluorescence intensity after the PTPA-QM (10 μM) was incubated with various analytes in the DMSO and (DMSO:glycerol = 20:80, *v*/*v*) for 10 min (1) PTPA-QM (2) Ca^2+^ (3) Fe^3+^ (4) K^+^ (5) Na^+^ (6) F^−^ (7) Cl^−^ (8) Br^−^ (9) I^−^ (10) SO_3_^2−^ (11) CO_3_^2−^ (12) HSO_3_^−^ (13) H_2_O_2_ (14) GSH (15) TrP (16) Phe (17) Met (18)Thr (19) Glu (20) Lys. λem = 385/615 nm.

To examine the performance of PTPA-QM, its photostability was investigated. The fluorescence intensity of PTPA-QM at 615 nm was recorded by continuous laser irradiation for 16 min after dissolving the PTPA-QM in different levels of glycerol solution (0%, 25%, 50%, 75%, 99%). As shown in Figure 3a, the fluorescence intensity of the fluorescent probe PTPA-QM hardly changed in different concentrations of glycerol, and the probe exhibited good photostability. 

A fluorescent probe with specificity and selectivity for the test article compared to potential competing species is a necessary condition for a good probe. We evaluated the response of PTPA-QM to various biologically relevant species including metal ions, anions, and amino acids in different solutions (DMSO, DMSO: glycerol = 1:4). The fluorescence intensity of the fluorescent probe PTPA-QM was almost unchanged (Figure 3b). Including common amino acids exhibited no obvious effect on the emission of PTPA-QM. The results show that PTPA-QM has a negligible effect on factors other than viscosity sensitivity. Therefore, PTPA-QM can be used as an ideal fluorescent probe for viscosity detection.

**Figure 4:**
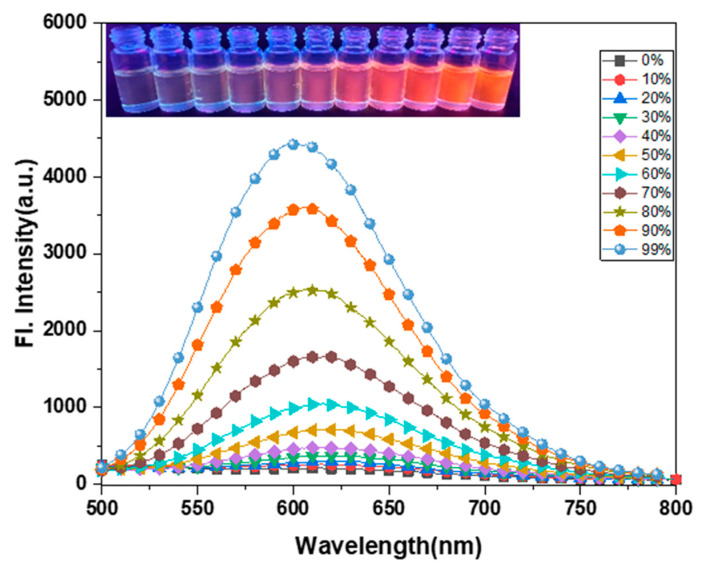
Fluorescence emission spectra of PTPA-QM in mixed DMSO-glycerol solutions with different glycerol volume fractions (10 μM). Inset: fluorescence photographs of the compounds in glycerol-DMSO mixtures with different glycerol volume fractions under 365 nm irradiation.

Subsequently, we explored the fluorescence response pattern of PTPA-QM in the DMSO-glycerol system. As shown in Figure 4, the fluorescence intensity of PTPA-QM at 615 nm was negligible in the DMSO. With the increasing content of glycerol, the viscosity of the detection system also increased gradually, and its fluorescence intensity increased significantly. The fluorescence intensity of PTPA-QM in 99% glycerol solution was 22-fold higher than that in pure DMSO. 

**Figure 5:**
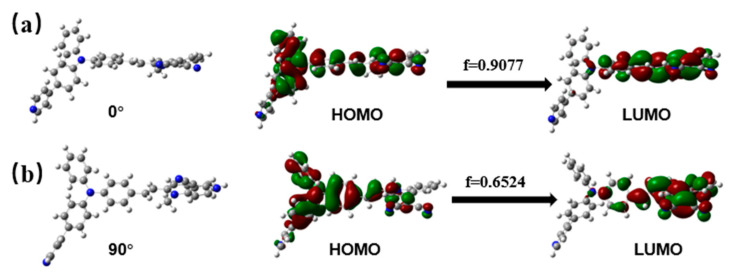
The optimized structure, HOMO, LUMO, and oscillator strength of PTPA-QM at around (**a**) 0° and (**b**) 90°.

The formation of fluorescence differences in PTPA-QM in different viscosities can be demonstrated by time-dependent density functional theory (TD-DFT) calculations using the B3LYP/6-311G method of the Gaussian 09 package. In the ground state, the triphenylamine group and quinoline–malononitrile group remain almost planar. The oscillator strength (ƒem) was 0.9077 (Figure 5). However, when the fluorescent molecule is excited, the triphenylamine group and quinoline–malononitrile groups are in a perpendicular position. The conformation of NIR-PF was in a distorted state, and the oscillator strength (ƒem) was 0.6524. It was demonstrated that in viscous solutions, the fluorescent molecules undergo the TICT process. Meanwhile, the HOMO of PTPA-QM primarily resided on the triphenylamine group, while the LUMO predominantly resided on the quinoline–malononitrile, thus forming the typical TICT process. These results indicate that the ground state of the fluorescent probe PTPA-QM can be excited to an excited state when under glycerol conditions, thus leading to an intense fluorescence. However, PTPA-QM can rotate freely in a low-viscosity environment, forming a distorted excited state, which leads to weak fluorescence. 

Based on the spectral data, a possible response mechanism of PTPA-QM to viscosity was proposed. In a low-viscosity environment, rotations between free-orbiting electron donors and electron acceptors are allowed. When the fluorescent probe is excited by photons, the excited state energy is released non-radiometrically by reversing the intramolecular charge transfer, showing weak fluorescence. In a high-viscosity solution environment, the intramolecular rotation of the fluorescent probe is inhibited. The excitation energy is released in the form of radiation, which eventually leads to a stronger fluorescence.

### 3.3. Cell Imaging

**Figure 6:**
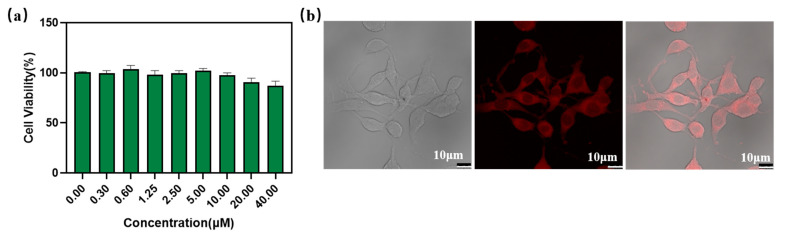
(**a**) PTPA-QM cytotoxicity assays at different concentrations of PC12 cells (horizontal coordinate: concentration/ μM) (**b**) CLSM in PC12 cells by PTPA-QM (10.0 μM).

To study the latent function values of PTPA-QM towards biological systems, we carried out fluorescent bio-imaging experiments in PC12 cells. The cytotoxicity of PTPA-QM against PC12 cells was assessed using CCK8 assays. Figure 6 indicated that the low concentration probe PTPA-QM (40 μM) showed low toxicity to cells and could be well applied to a biological application. Therefore, the PC12 cells were incubated into the culture plate and were cultured with the probe PTPA-QM (10 μM) for about 1 h and imaged through CLSM.

### 3.4. Specificity of PTPA-QM to Aβ Plaques in Brain Slices

The active cavity sites of amyloid fibrils were obtained by simulation with Schrödinger software and then docked with the structurally optimized PTPA-QM to obtain the interaction model of PTPA-QM with amyloid fibrils. As can be seen from the figure, the space of the active cavity of amyloid fibrils is very narrow, making it difficult for the highly distorted structure to insert into the active cavity. The linearly structured fluorescent probe PTPA-QM has a very small rigid backbone structure that is easily inserted between the β-folded lamellar structures of the amyloid fibrils and forms interactions with the amino acid residues on the amyloid fibrils.

**Figure 7:**
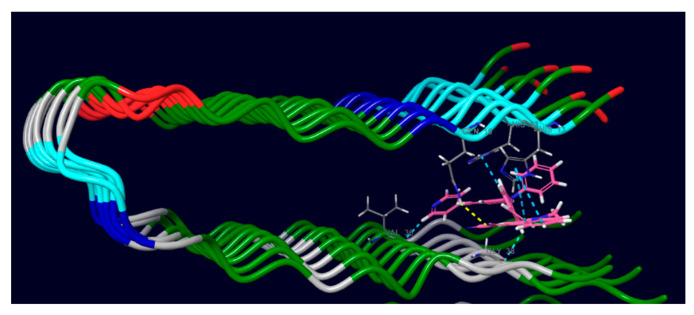
Binding models of PTPA-QM to amyloid fibrils obtained by Schrödinger software calculations.

Wang et al. [37] found that the molecules of ThT could insert into the β-folded lamellae of Aβ fibrils. ThT only showed a weak N-H∙∙∙π interaction with the side groups of HIS13. The molecules of PTPA-QM could be inserted into the β-folded lamellae of Aβ fibrils and interact with the amino acid residues of HIS13, VAL36, GLY38, and GLN15 (Figure 7), while PTPA-QM possessed strong N-H∙∙∙O, C-H∙∙∙O, N-H∙∙∙π, π∙∙∙π, N+∙∙∙π multiple interactions. Through these intermolecular interactions, the probe is subject to intramolecular motion and emits a strong fluorescence when excited by light, thus enabling the fluorescence detection of Aβ fibers.

The sensitivity of the compound to Aβ was investigated by the fluorescence detection of PTPA-QM on Aβ. As an AIE probe, PTPA-QM showed essentially a weak fluorescence response. However, when a small amount of Aβ fiber solution was added, the fluorescence intensity increased. However, the change in the fluorescence intensity increased only about two-fold. After binding to the Aβ fiber, the maximum emission peak of the compound PTPA-QM was located at 625 nm and showed red fluorescence (Figure S2). The most important reason is that the binding of PTPA-QM to Aβ aggregates limits the free rotation of the bonds, which enhances the fluorescence emission. We investigated the selectivity of this probe PTPA-QM (PTPA-QM,10 μM, ethanol/H_2_O = 4:6; proteins, 10 μM). Several proteins were selected for fluorescence detection. For example, Aβ aggregates, the aggregation of amylin, the aggregation of a-synuclein, and amyloid fibrils from hen egg white lysozyme (Figure S3). However, when other proteins were added, there was a slight change in the fluorescence intensity. Therefore, the fluorescent probe PTPA-QM is less selective for Aβ aggregates. Therefore, animal tissue sections were selected for staining in the imaging experiments.

The main pathological feature of AD is the abnormal deposition of Aβ in the brain. Therefore, the detection of Aβ in brain tissue is important for the detection and pathological study of AD. Neuroinflammatory regulation and Aβ production are key factors in the pathogenesis of AD. Therefore, Tg mice and LPS-induced inflammatory cognitive impairment mice were selected for the applied study of fluorescent molecules.

**Figure 8:**
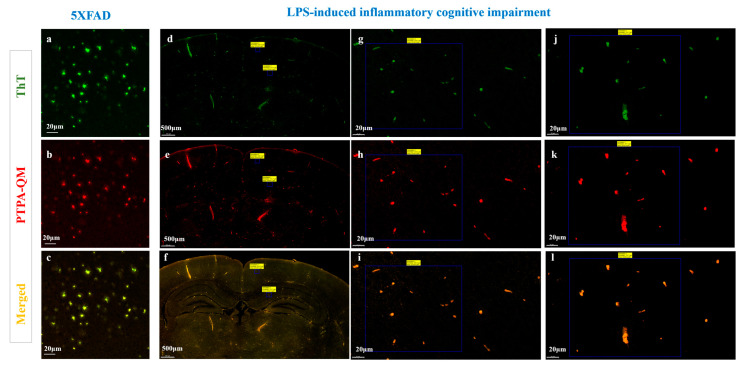
Colocalization of ThT labeling (**a**,**d**,**g**,**j**) and PTPA-QM (**b**,**e**,**h**,**k**) with immunostaining of Aβ in the brain sections of 5XFAD transgenic mice (**a**–**c**) and LPS-induced inflammatory cognitive impairment mice (**d**–**l**). (**g**,**h**,**i**) Cerebral cortex; (**j**,**k**,**l**) hippocampus. (**c**,**f**,**i**,**l**) are the merged images of PTPA-QM and ThT.

First, brain tissue sections from Tg mice (5XFAD, 9 months old, male) were stained for the neuropathological fluorescence co-localization of Aβ using the probe PTPA-QM to assess the targeting of the probe to Aβ. Fluorescence co-localization imaging results showed that many fluorescent patches could be observed in brain tissue sections of Tg mice co-incubated with ThT and PTPA-QM. The superimposed images showed that the green spots of the ThT-stained image overlapped almost completely with the red spots of the PTPA-QM-stained image (Figure 8a–c). In LPS-induced inflammatory cognitive impairment mice (C57BL/6J mice, 3 months old, male), fluorescent molecules were co-stained with ThT on brain sections in the experiments. We can see that the plaques stained with the probe PTPA-QM are consistent with ThT (Figure 8d–l). Additionally, it can rapidly stain Aβ within 10 min in mouse brain sections. Therefore, it is clear from the experimental results that the probe PTPA-QM can bind to Aβ in the brain and fluoresce at excitation wavelengths.

## 4. Conclusions

In summary, we developed a fluorescent probe, PTPA-QM, using a simple synthetic procedure. PTPA-QM shows a good performance for viscosity with enhanced fluorescence emission at 615 nm. The PTPA-QM exhibits a significant AIE effect, with significant turn-on fluorescence enhancement. Additionally, this probe has low cytotoxicity to PC12. In addition, PTPA-QM was successfully used for the fluorescence imaging of Aβ in the brain of Tg mice and LPS-induced inflammatory cognitive impairment mice. PTPA-QM rapidly stains Aβ in mouse brain sections in less than 10 min. Overall, all these studies suggest that our AIE probe holds great promise in exploring the pathology of AD, and, with further development, could be widely used for early diagnosis.

## Data Availability

Not applicable.

## Supplementary Materials

The following supporting information can be downloaded at: https://www.mdpi.com/article/10.3390/bios13060610/s1, Figure S1: Normalized absorption spectra of PTPA-QM (1.0 × 10^−5^ M) (black). Normalized FL spectra of of PTPA-QM (1.0 × 10^−5^ M) (red); Figure S2: Fluorescent responses of PTPA-QM to the aggregates of fibrils. (Ethanol/H_2_O = 4:6); Figure S3: Fluorescent responses of PTPA-QM to proteins. (PTPA-QM,10 μM, ethanol/H_2_O = 4:6; proteins, 10 μM); Figure S4: ^1^ H NMR, ^13^ C NMR and HR-MS spectra (PTPA-QM)
