# Supplementary material for: A Novel Aggregation-Induced Emission Fluorescent Probe for Detection of β-Amyloid Based on Pyridinyltriphenylamine and Quinoline–Malononitrile
Source: Biosensors (Basel). 2023 Jun 2;13(6):610. doi: 10.3390/bios13060610 (PMC10296659; doi:10.3390/bios13060610)
Supplement: Supplementary file 1 [file biosensors-13-00610-s001.zip › biosensors-2350872-supplementary.pdf]

biosensors

Supplementary Materials

A Novel Aggregation-Induced Emission Fluorescent Probe for Detection of β-Amyloid Based on Pyridinyltriphenylamine and Quinoline–Malononitrile

Yan Fang, Qi wang, Chenlong Xiang, Guijin Liu * and Junjian Li *

1. Experimental Method

PC12 cells were cultured in RPMI 1640 supplemented with 10% FBS and 1% penicillin–streptomycin at 37 °C in a humidified incubator with 5% CO_2. PC12 cells were inoculated at a density of 1 × 10^5 cells/mL in a 24-well plate with crawlers and incubated at 37 °C for 24–48 h. After more than 50% of the cells on the crawlers were covered, the original medium was aspirated from each well. Then, we gently rinsed the dead cells and debris from the surface of the crawl plate with PBS buffer and repeated twice. After washing, 1 mL of RPMI 1640 medium containing 10 μM probe was added to each well under light-proof conditions. Then, 24-well plates were gently shaken after the addition and were incubated in a cell culture incubator for 1 h. After 1 h, the plates were removed and the culture medium was aspirated from the wells, and each well was washed three times with PBS buffer to remove the residual probe from the crawler. After that, 1 mL of paraformaldehyde was added to all wells to fix the cells for 15 min, and paraformaldehyde was aspirated off after fixation and they were then washed with PBS buffer 3 times. We then took a clean slide, placed a drop of anti-fluorescence quencher in its center, carefully clipped out the above treated cell crawl with forceps, and slowly covered the long cell side with anti-fluorescence quencher.

The cells were cultivated as above. PC12 cells were grown in 96-well plates at a density of 1 × 10^5 cells/mL. Probe solutions of 0.3 μM, 0.6 μM, 1.25 μM, 2.5 μM, 5 μM, 10 μM, 20 μM, and 40 μM were obtained, respectively. The cells were then removed from the incubator and the previous cell culture solution was aspirated. Then, we proceeded to add 100 μL of the above concentration of the probe solution to each well. Then we set up three parallel wells for each concentration and set up a blank control group. After all of the additions, the 96-well plates continued to be incubated in a cell culture incubator for 24 h. After 24 h of cell culture in the 96-well plates, the previous cell culture solution was aspirated. After 24 h of incubation, the cells were removed from the 96-well plates, and 100 μL of CCK-8 was added to each well under light-proof conditions, and the plates were placed in the cell culture incubator for 1 h. After incubation, the absorbance values at 480 nm were measured directly using an ELISA.

Tg mice (5XFAD, 9 months old, male) brain tissue sections were presented by Mr. Wang Yalong from the School of Biomedical Engineering, Hainan University.

Clean-grade C57BL/6J mice (3 months old, male) weighing between 20 and 2 g were used in the experiments and were purchased from Hunan Sleek Jingda Company (animal permit number SCXK Xiang 2019-0004). The mice were placed in a pathogen-free facility at the Animal Feeding Center of Hainan Medical College and maintained in a 12-hour light–dark cycle with free access to standard food and water. This experiment was approved by the Laboratory Animal Management Committee of Hainan University (HNUAUCC-2021-00020). All animal operations followed the guidelines for the protection and use of laboratory animals and the management of laboratory animals at Hainan University.

C57BL/6J clean-grade male mice were randomly assigned into 3 groups (n = 12/group): control group (Control) and LPS model group (LPS). The model group was

given an intraperitoneal injection of 1 mg/kg/day of LPS (Beijing Solaibao Technology Co., Ltd.). Mice in the Control group were injected intraperitoneally with the same volume of saline. The mice were fasted overnight for 24 h after the last treatment. Apical perfusion was performed with pre-chilled PBS (pH 7.4), brain tissue was extracted, and the extracted brain tissue was placed in 4% paraformaldehyde solution for storage and fixation for the next experiments.

Paraformaldehyde fixed brain tissue was used for in vitro wash imaging of Aβ plaques. The brain sections were washed with PBS (10 mM, pH = 7.4) 3 times and then incubated with ThT (2 μM) for 3 min. The staining brain sections wash with DMSO:H_2O = 1:1 3 times. The brain sections were incubated with a fluorescent probe PTPA-QM (2 μM) in DMSO for 3 min. The staining brain sections were washed with DMSO:H_2O = 1:1 3 times before packaging. The excitation wavelength employed on ThT is 405 nm, and the corresponding collected wavelength is 525 ± 25 nm. As for PTPA-QM, the excitation wavelength is 488 nm, and the corresponding collected wavelength is 600 ± 40 nm.

Calculation of the fluorescence quantum yield (QYs): The QYs of the probe PTPA-QM in different solvents was calculated and measured using fluorescein as the reference substance. The absorbance of the probe in THF and glycerol was first measured to ensure that the absorbance of the probe was less than 0.05. Then, all the systems to be measured were tested for their fluorescence spectra. The fluorescence quantum yields were calculated as follows:

$$\emptyset_S = \emptyset_R \frac{F_R}{F_S}\frac{A_S}{A_R}\left(\frac{n_S}{n_R}\right)^2$$

where S and R represent the substance to be measured and the reference material (FITC: Fluorescein isothiocyanate), respectively. $\emptyset$ represents the fluorescence quantum yield; F is the integrated fluorescent intensity; and A is the absorbance at the excitation wavelength n which represents the refractive index of the solvent.

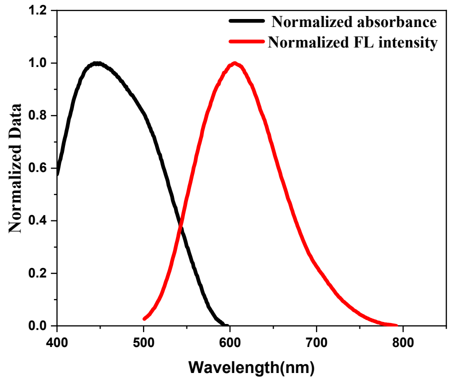

Figure S1. Normalized absorption spectra of PTPA-QM (1.0 × 10^{-5} M) (black). Normalized FL spectra of of PTPA-QM (1.0 × 10^{-5} M) (red).

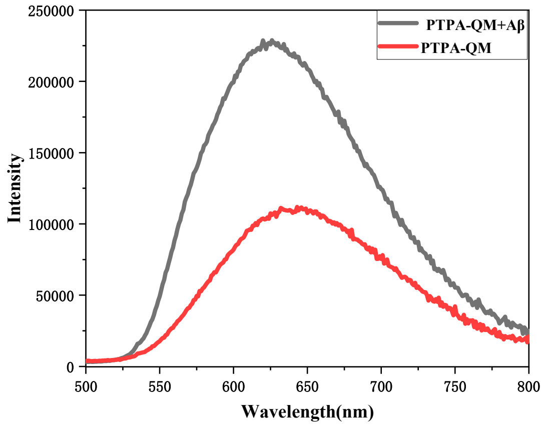

Figure S2. Fluorescent responses of PTPA-QM to the aggregates of fibrils. (Ethanol/H_2O = 4:6).

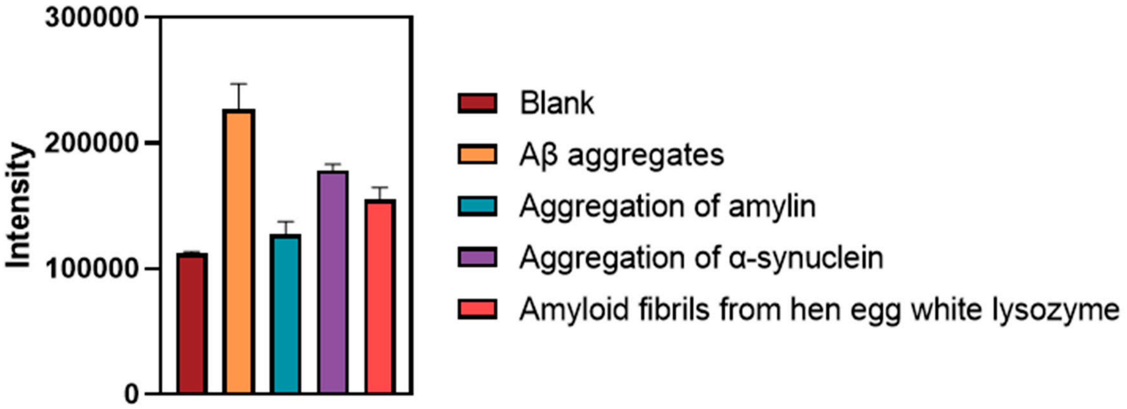

Figure S3. Fluorescent responses of PTPA-QM to proteins. (PTPA-QM,10 μM, ethanol/H_2O = 4:6; proteins, 10 μM).

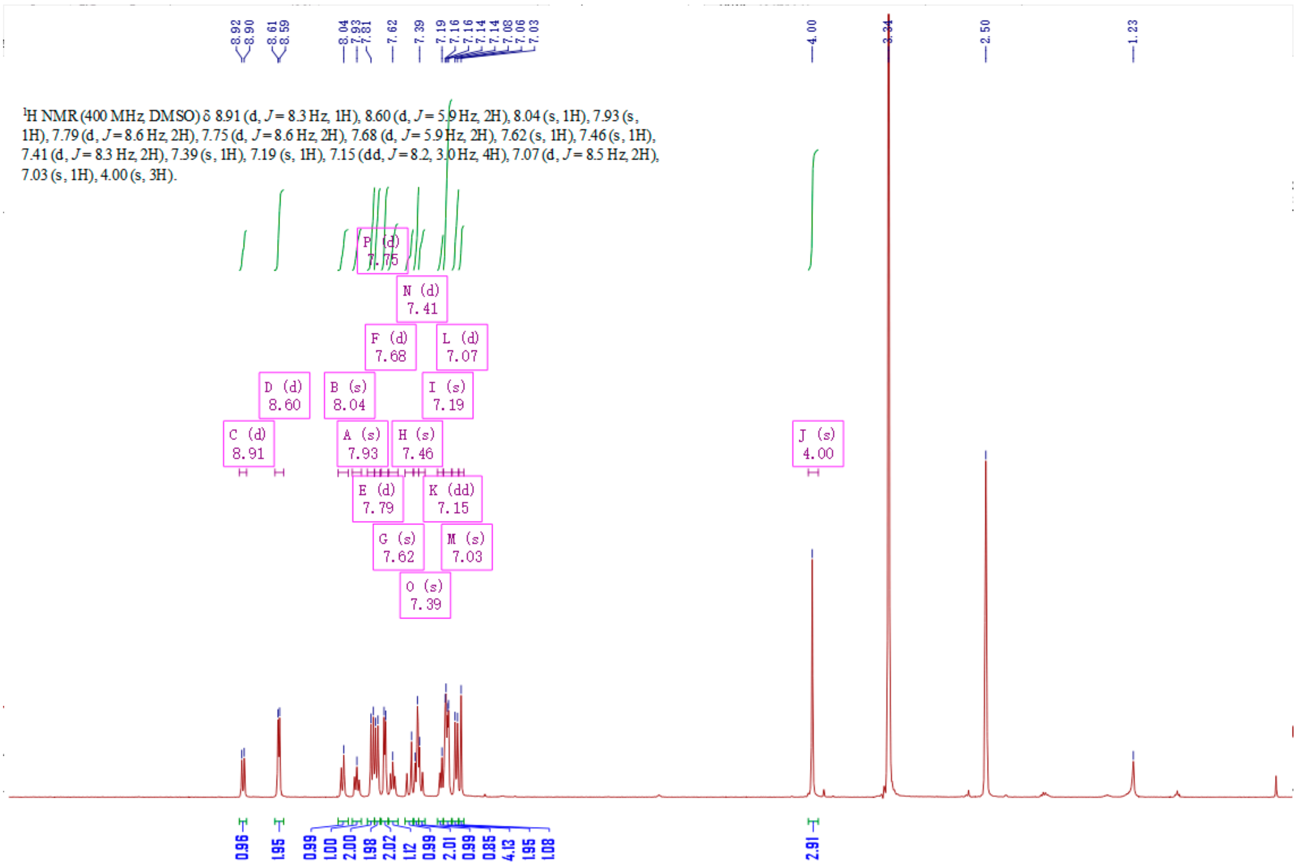
1H NMR (400 MHz, DMSO) δ 8.91 (d, J = 8.3 Hz, 1H), 8.60 (d, J = 5.9 Hz, 2H), 8.04 (s, 1H), 7.93 (s, 1H), 7.79 (d, J = 8.6 Hz, 2H), 7.75 (d, J = 8.6 Hz, 2H), 7.68 (d, J = 5.9 Hz, 2H), 7.62 (s, 1H), 7.46 (s, 1H), 7.41 (d, J = 8.3 Hz, 2H), 7.39 (s, 1H), 7.19 (s, 1H), 7.15 (dd, J = 8.2, 3.0 Hz, 4H), 7.07 (d, J = 8.5 Hz, 2H), 7.03 (s, 1H), 4.00 (s, 3H).

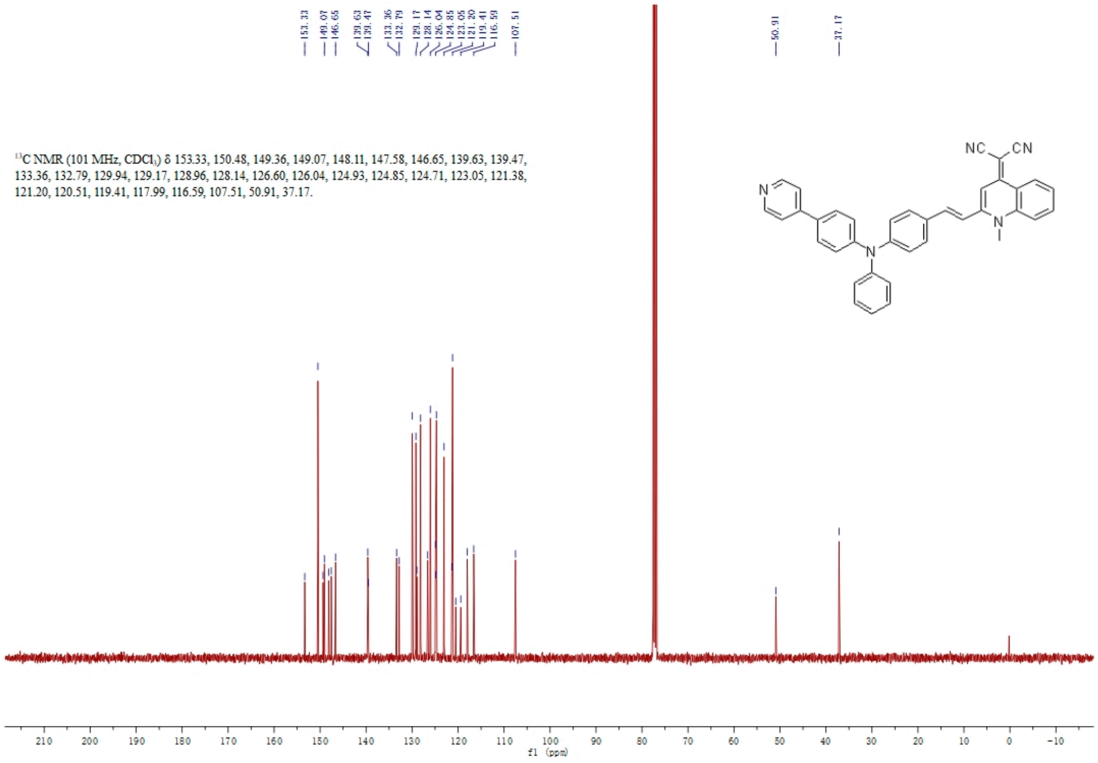
13C NMR (101 MHz, CDCl3) δ 153.33, 150.48, 149.36, 149.07, 148.11, 147.58, 146.65, 139.63, 139.47, 133.36, 132.79, 129.94, 129.17, 128.96, 128.14, 126.60, 126.04, 124.93, 124.85, 124.71, 123.05, 121.38, 121.20, 120.51, 119.41, 117.99, 116.59, 107.51, 50.91, 37.17.
NC
CN
N
N
N
f1 (ppm)

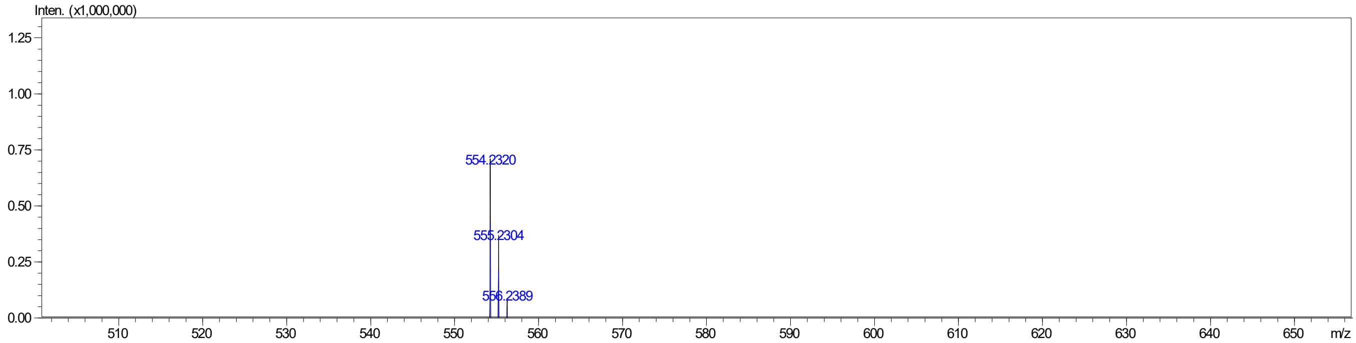

Figure S4. ^{1}H NMR, ^{13}C NMR and HR-MS spectra (PTPA-QM).

Disclaimer/Publisher's Note: The statements, opinions and data contained in all publications are solely those of the individual author(s) and contributor(s) and not of MDPI and/or the editor(s). MDPI and/or the editor(s) disclaim responsibility for any injury to people or property resulting from any ideas, methods, instructions or products referred to in the content.
